# CNTF Induces Regeneration of Cone Outer Segments in a Rat Model of Retinal Degeneration

**DOI:** 10.1371/journal.pone.0009495

**Published:** 2010-03-02

**Authors:** Yiwen Li, Weng Tao, Lingyu Luo, Deqiang Huang, Konrad Kauper, Paul Stabila, Matthew M. LaVail, Alan M. Laties, Rong Wen

**Affiliations:** 1 Bascom Palmer Eye Institute, Miller School of Medicine, University of Miami, Miami, Florida, United States of America; 2 Neurotech USA, Lincoln, Rhode Island, United States of America; 3 Beckman Vision Center, University of California San Francisco, San Francisco, California, United States of America; 4 Department of Ophthalmology, School of Medicine, University of Pennsylvania, Philadelphia, Pennsylvania, United States of America; Emory University, United States of America

## Abstract

**Background:**

Cone photoreceptors are responsible for color and central vision. In the late stage of retinitis pigmentosa and in geographic atrophy associated with age-related macular degeneration, cone degeneration eventually causes loss of central vision. In the present work, we investigated cone degeneration secondary to rod loss in the S334ter-3 transgenic rats carrying the rhodopsin mutation S334ter.

**Methodology/Principal Findings:**

Recombinant human ciliary neurotrophic factor (CNTF) was delivered by intravitreal injection to the left eye of an animal, and vehicle to the right eye. Eyes were harvested 10 days after injection. Cone outer segments (COS), and cell bodies were identified by staining with peanut agglutinin and cone arrestin antibodies in whole-mount retinas. For long-term treatment with CNTF, CNTF secreting microdevices were implanted into the left eyes at postnatal day (PD) 20 and control devices into the right eyes. Cone ERG was recorded at PD 160 from implanted animals. Our results demonstrate that an early sign of cone degeneration is the loss of COS, which concentrated in many small areas throughout the retina and is progressive with age. Treatment with CNTF induces regeneration of COS and thus reverses the degeneration process in early stages of cone degeneration. Sustained delivery of CNTF prevents cones from degeneration and helps them to maintain COS and light-sensing function.

**Conclusions/Significance:**

Loss of COS is an early sign of secondary cone degeneration whereas cell death occurs much later. At early stages, degenerating cones are capable of regenerating outer segments, indicating the reversal of the degenerative process. Sustained delivery of CNTF preserves cone cells and their function. Long-term treatment with CNTF starting at early stages of degeneration could be a viable strategy for preservation of central vision for patients with retinal degenerations.

## Introduction

Retinal degenerations are a major cause of blindness for which no effective treatments are available [1]. It is estimated that one in 3,500 to 4,000 people are affected by retinitis pigmentosa (RP), a heterogeneous group of inherited retinal degenerative disorders. Mutations in at least 34 genes are identified to be responsible for RP [1]. Most mutations responsible for the RP phenotype selectively affect rod photoreceptors, nevertheless cones undergo degeneration secondary to the loss of rods, often leading to total blindness [1], [2]. How to save the cones has become a major research challenge.

A number of neurotrophic factors have demonstrated neuroprotective effects in mitigating retinal degenerations [3]. Among them, (CNTF ciliary neurotrophic factor) has shown a promising capability to inhibit progression of retinal degenerations in a variety of animal models, as well as in light-induced damage [4]–[12]. CNTF, initially identified as a factor in chick embryo extract that supported viability of embryonic chick ciliary neurons [13], [14] and later purified to homogeneity [15], [16], exhibits multiple biological effects in the retina. CNTF promotes the survival and axonal regeneration of retinal ganglion cells [17]–[20] and acts as neuronal differentiation factor, promotes the differentiation of a subclass of cone photoreceptors expressing green opsin in the developing chick retina [21]–[23], enhances the expression of bipolar neuron markers in rat retinal cultures [24]–[26], and inhibits rod photoreceptor cell differentiation [22], [25]–[27], and promotes Müller glia genesis from the postnatal retinal progenitor pool [28]. It has been reported that CNTF regulates the phototransduction machinery of rod photoreceptors, and the regulation was mediated through Müller cells [29], [30].

In the present study, we investigated the secondary degeneration of cones in transgenic rats carrying the rhodopsin mutation S334ter, a model of retinal degeneration. In this model, rod photoreceptors undergo rapid degeneration soon after birth [31]. Cone degeneration starts at the peak of rod degeneration, and is characterized by loss of COS in many round or irregularly shaped areas across the retina. When treated with CNTF, cones are stimulated to regenerate their COS. These results provide evidence that in early stages, CNTF reverses secondary cone degeneration. In addition, our results show that sustained delivery of CNTF helps cones to maintain COS and their functions.

## Results

### Loss of COS in Early Stages of Cone Degeneration

**Figure 1:**
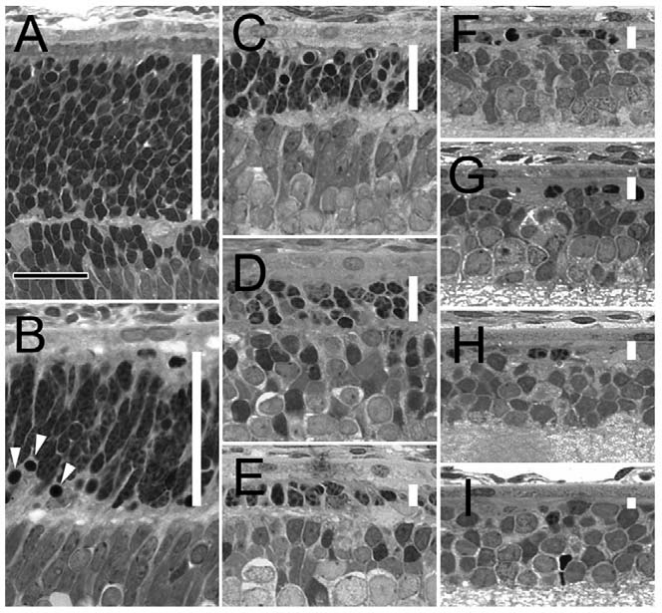
Photoreceptor degeneration in homozygous S334ter-3 rats. Plastic-embedded sections of retinas from S334ter-3 rats of PD 8 (A), 10 (B), 12 (C), 14 (D), 16 (E), 18 (F), 20 (G), 30 (H), and 60 (I) were examined by light microscopy (superior regions). The outer nuclear layer (ONL) is indicated in each panel by a white vertical bar. Pyknotic nuclei are indicated by white arrowheads (B). Loss of photoreceptors was progressive and by PD 20 most of rod photoreceptors were degenerated, and the ONL had less than a complete row of nuclei. Sections were stained with toluidine blue. Scale bar, 20 µm.

**Figure 2:**
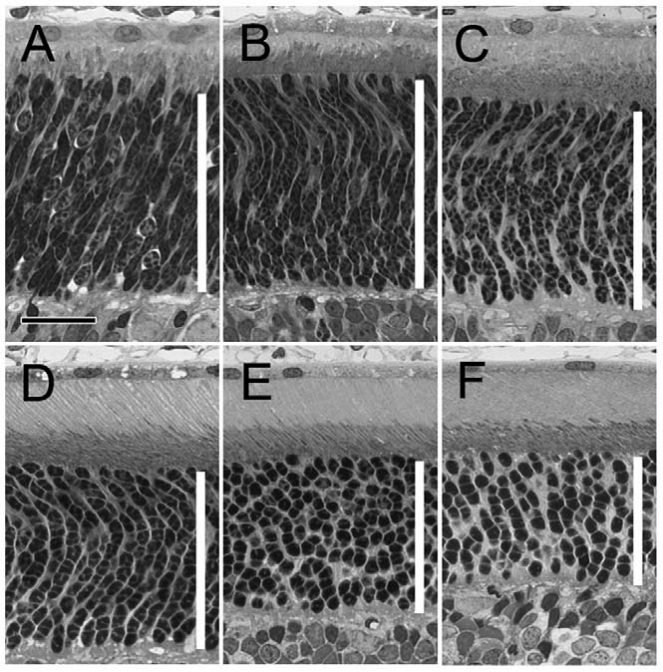
Photoreceptors in Sprague-Dawley rats. Plastic-embedded sections of retinas from wild-type Sprague-Dawley rats of PD 8 (A), 12 (B), 16 (C), 20 (D), 30 (E), and 60 (F) were examined by light microscopy (superior regions). The ONL is indicated in each panel by a white vertical bar. No progressive loss of photoreceptors is observed. Sections were stained with toluidine blue. Scale bar, 20 µm.

The degeneration rate of homozygous S334ter-3 rats (Fig. 1) is quite similar to that observed in the heterozygous animals [31]. Pyknotic nuclei are readily seen by postnatal day (PD) 10. Massive rod photoreceptor death occurs between PD 12 to PD 16. By PD 20, most of the rod photoreceptors are degenerated. Only one incomplete row of photoreceptor nuclei remains in the outer nuclear layer (ONL) where photoreceptor cell nuclei reside. By PD 30 and PD 60, even fewer nuclei remain and all appear to be cone nuclei. In contrast, no significant photoreceptor loss occurs in wild-type rats from PD 8 to PD 60 (Fig. 2).

**Figure 3:**
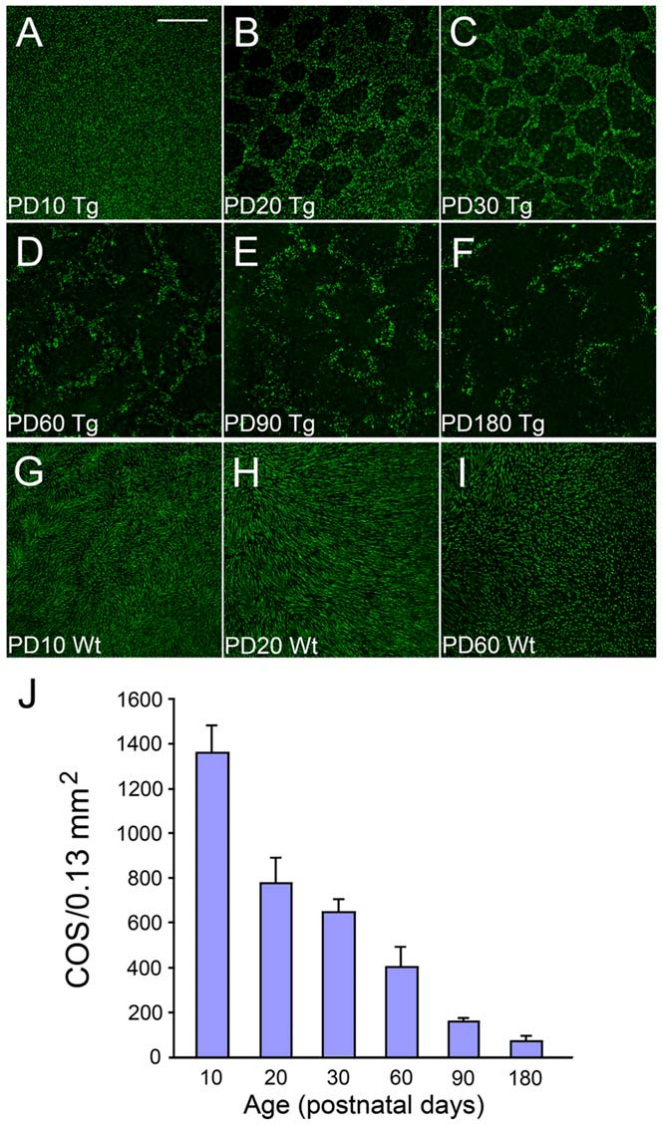
Loss of COS in the retinas of S334ter-3 rats. Whole-mounted retinas (superior regions) of S334ter-3 rats (Tg) are shown at PD 10 (A), 20 (B), 30 (C), 60 (D), 90 (E), and 180 (F), as well as retinas of wild-type (Wt) rats at PD10 (G), 20 (H), and 60 (I). Retinas were stained with PNA and examined by confocoal microscopy. In S334ter-3 retinas, loss of PNA staining is concentrated in small round or irregularly shaped areas. The loss of PNA staining was progressive with age (A–F). Quantitative analysis of the density of PNA positive cells in the S334-ter-3 retinas clearly shows the progressive loss of PNA positive cells in the superior retina (J, mean ± SD, n = 4). No such loss of PNA positive cells was found in wild-type retinas (G–I). Scale bar: 200 µm.

To investigate the distribution of COS, we used PNA to identify COS in whole-mounted retinas. PNA specifically binds to the cone photoreceptor extracellular matrix and stains COS [32], [33]. At PD 10, PNA staining is evenly distributed across the entire retina (Fig. 3A). Loss of PNA staining first is detected at PD12 when rod degeneration is at its peak (data not shown). By PD 20, many small round or irregularly shaped PNA-negative areas were observed, indicating focal loss of COS (Fig. 3B). The PNA staining outside the PNA-negative areas is similar to the retina of PD 10. The loss of PNA staining progresses with age (Fig. 3A–3F), as demonstrated clearly in the quantitative analysis of COS densities (Fig. 3J). In comparison, PNA staining in wild-type animals was distributed evenly without any PNA-negative area (Fig. 3G–3I).

**Figure 4:**
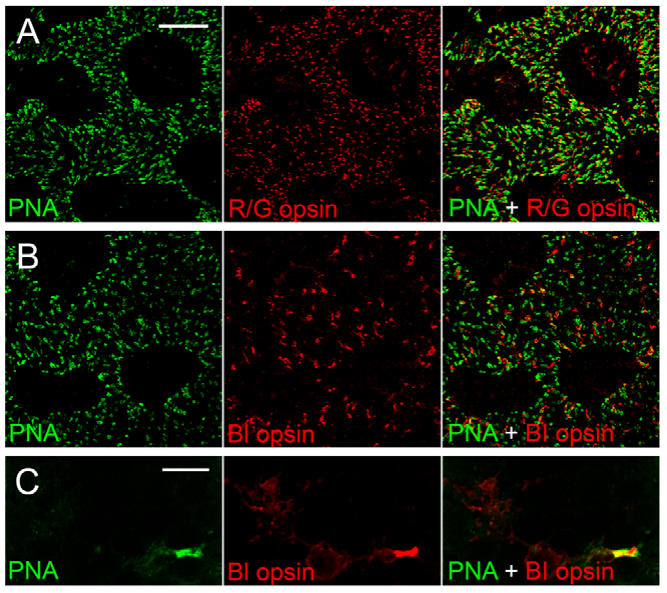
Specificity of PNA staining to COS. Whole-mounted retinas (superior regions) of S334ter-3 rats (PD 30) were double stained with PNA and antibodies against red/green opsin (A) or blue opsin (B). PNA-staining overlaps with red/green opsin or blue opsin staining very well. The specificity of PNA staining to COS was confirmed under high magnification (C). PNA stained almost exclusively COS (C), whereas antibodies against blue opsin stained not only COS, but also, to a lesser degree, the inner segment, the cell body, and the synaptic terminal (C). Scale bar: 50 µm for A and B; 10 µm for C.

To confirm the specificity of PNA staining of COS, we performed double-staining experiments with PNA and antibodies against red/green or blue opsin (Fig. 4). The distribution of red/green (Fig. 4A) or blue opsin (Fig. 4B) immunostaining overlaps very well with PNA staining. The specificity of PNA staining of COS is clearly shown at higher magnification in figure 4C in which a cone photoreceptor was double-stained with PNA and antibodies against blue opsin. PNA labeled almost exclusively the outer segment (Fig. 4C), whereas antibodies against blue opsin stained not only the outer segment, but also, to a lesser degree, the inner segment, the cell body, and the synaptic terminal (Fig. 4C).

**Figure 5:**
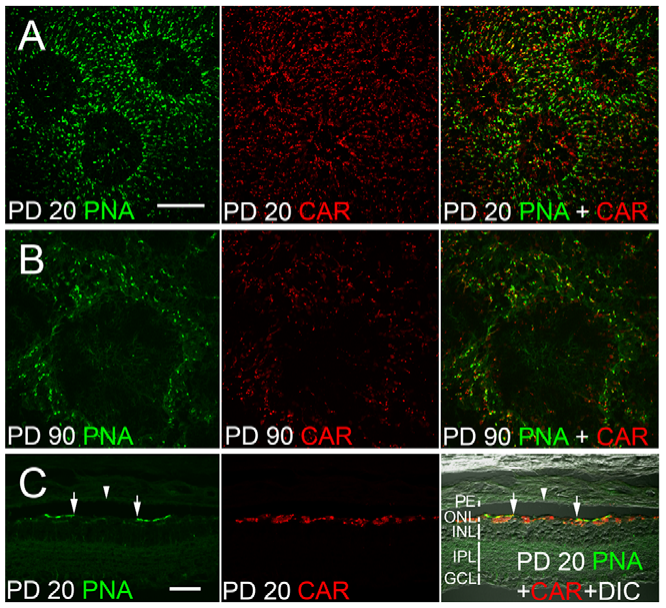
Loss of COS and cone cells. Whole-mounted retinas (superior regions) of S334ter-3 rats were double stained with PNA and antibodies against cone arrestin (CAR). In the retina of PD 20 (A), many CAR positive cells were found in the PNA-negative areas. In the retina of PD 90 (B), however, only a few CAR-positive cells remained in the PNA-negative areas. Cryosections of a PD 20 retina (C) double stained with PNA and antibodies against CAR showed a gap of PNA staining corresponding to a negative area seen in whole-mounted retinas (C, between two arrows). CAR positive cells were found in the gap. The retina detachment (arrowhead) showed that in the PNA-negative area (between two arrows), the corresponding RPE side is also negative. Retinal layers were indicated in C with white vertical bars. PE: RPE; ONL: outer nuclear layer; INL: inner nuclear layer; IPL: inner plexiform layer; GCL, ganglion cell layer. Scale bars: 100 µm for A and B; 50 µm for C.

We next stained cones using antibodies against cone arrestin (CAR), which specifically stained cone cells from the synaptic terminals to the outer segment [34]. At PD 20, many CAR-positive cells were found inside PNA-negative areas (Fig. 5A), identifying them cones without COS. By PD 90, however, only a few CAR-positive cells remained in the PNA-negative areas (Fig. 5B). Thus, by PD 90, most of the cones in the PNA-negative areas were degenerated. Staining in retinal sections showed that at PD 20, PNA labeling was interrupted by PNA-negative gaps (Fig. 5C, between two arrows), corresponding to the PNA-negative areas found in whole-mounted retinas. Many CAR-positive cells were found inside the PNA-negative gaps (Fig. 5C), consistent with data from whole-mounted retinas.

In addition, data from retinal sections provided evidence that PNA-negative areas in the whole-mounted retinas were not an artifact that might have been resulted from uneven separation of the retina from the RPE during tissue preparation. In a retinal detachment created during tissue preparation, the retina was negative of PNA staining, and so was the overlaying RPE (Fig. 5C, arrowheads). If the PNA-negative areas were an artifact of uneven tissue separation, the RPE side would have been PNA positive. This finding is consistent with results from additional experiments in which both the retinas and the RPE-choroid preparations were stained with PNA, but no PNA staining was detected in the RPE-choroid preparations (data not shown).

### CNTF Promotes COS Regeneration

**Figure 6:**
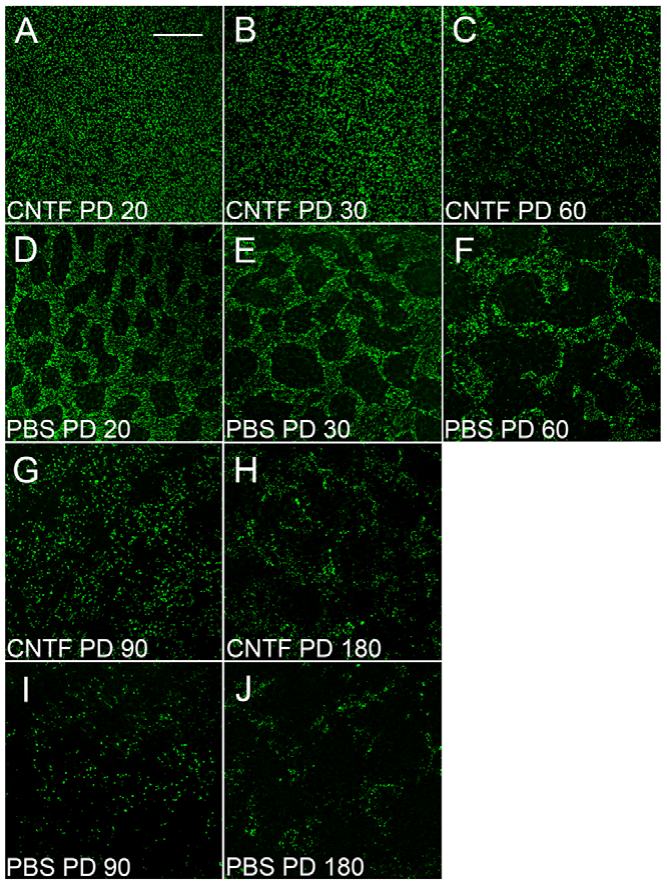
Increase in PNA-positive cells after CNTF treatment. Retinas (superior regions) of PD 10, 20, 50, 80, and 170 S334ter-3 rats were treated with either CNTF (left eyes) or PBS (right eyes) and collected 10 days after treatment. Whole-mounted retinas of PD 20 (i.e., injected at PD 10; A, CNTF; D, PBS), PD 30 (B, CNTF; E, PBS), PD 60 (C, CNTF; F, PBS), PD 90 (G, CNTF; I, PBS), and PD 180 (H, CNTF; J, PBS), were stained with PNA. Many PNA-negative areas appeared in PBS treated retinas, whereas no PNA-negative areas were seen in the PD 20 retinas treated with CNTF (A). The PNA-negative areas became much smaller and in most cases completely disappeared in PD 30 and 60 retinas treated with CNTF (B and C). Increase in PNA positive cells after CNTF treatment became less dramatic in PD 60 retinas (G), and by PD 180 (H), little or no significant increase is observed. Scale bar: 200 µm.

To investigate the effects of CNTF on cone photoreceptors, retinas were treated with CNTF and examined 10 days later. When treated with CNTF at PD 10 (endpoint PD 20), PNA staining was evenly distributed without obvious PNA-negative areas (Fig. 6A), whereas in PBS-treated fellow eyes, many PNA-negative areas appeared (Fig. 6D). When treated between the ages of PD 20 to PD 50 (endpoints PD 30 to PD 60), the PNA-negative areas became much smaller and in most cases completely disappeared (Fig. 6B and 6C). In contrast, many PNA-negative areas were found in the PBS-treated fellow retinas (Fig. 6E and 6F). The CNTF induced increase in PNA staining became less dramatic at PD 80 (endpoint PD 90, Fig. 6G) and almost no such increase was seen in retinas of PD 170 (endpoint PD 180, Fig. 6H), similar to PBS treated fellow retinas (Fig. 6I and 6J, respectively).

**Figure 7:**
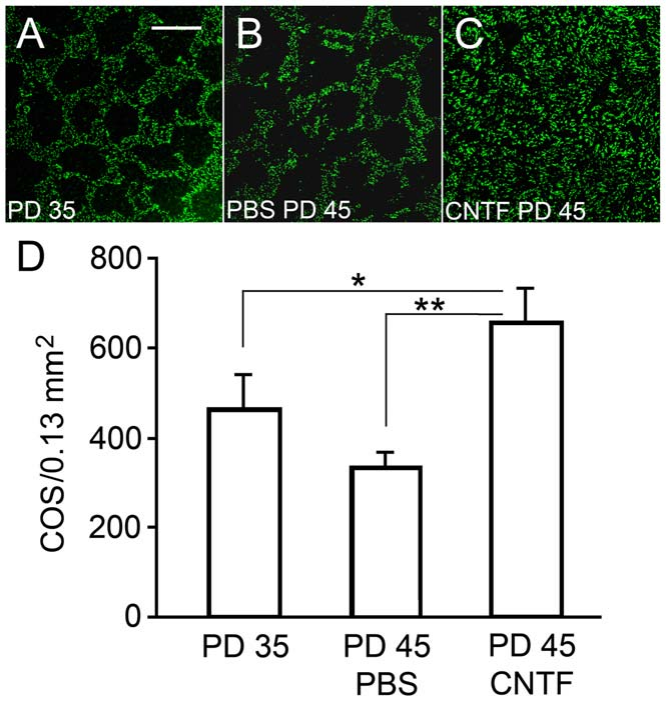
Regeneration of COS induced by CNTF treatment. Retinas (superior regions) of S334ter-3 rats at PD 35 were treated with either CNTF or PBS and harvested 10 days after treatment. Whole-mounted retinas of PD 35 (A, before treatment), PD 45 treated with PBS at PD 35 (B), and PD 45 treated with CNTF at PD 35 (C) were stained with PNA. PNA positive cells in CNTF treated retinas are more than those in either PBS treated retinas or PD 35 retinas. Quantitative analysis showed that PNA-positive cells are significantly more in the PD 45 CNTF treated retinas (657±76, n = 4) than in the PD 35 retinas (467±73, n = 3), or PD 45 retinas treated with PBS (334±33, n = 3) (D, mean ± SD), indicating regeneration of COS in response to CNTF treatment. Asterisk indicates *P*<0.05; double-asterisk indicates *P*≤0.01 (ANOVA analysis and Bonferroni test). Scale bar: 100 µm.

The increase in PNA-positive cells in PD 20 and PD 30 retinas treated with CNTF (Fig. 6B and 6C) indicates that CNTF treatment not only stopped further degeneration, but also induced reappearance of PNA staining, suggesting regeneration of COS. To confirm this observation, eyes were treated with CNTF at PD 35 and retinas were examined at PD 45 for PNA-positive cells. Indeed, the density of PNA-positive cells in CNTF treated retinas of PD 45 (Fig. 7C) was more than that in the retina of PD 35 (Fig. 7A) before the treatment. Quantitative analyses showed that the density of PNA-positive cells in CNTF treated retinas of PD 45 was significantly more than in the PD 35 controls when the treatment started (Fig. 7D). These results provide evidence that CNTF treatment can promote regeneration of COS in degenerating cones.

### Long-Term Effect of CNTF on Cones

**Figure 8:**
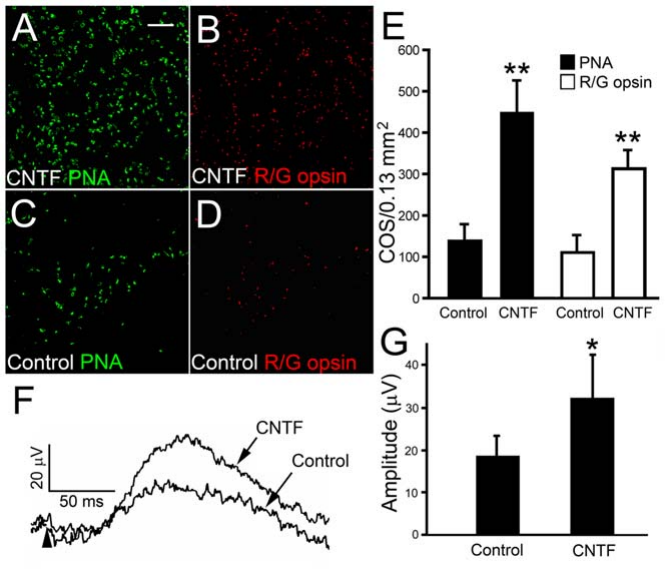
Long-term effects of sustained delivery of CNTF. Eyes of S334ter-3 rats were implanted with microdevices containing CNTF-secreting cells or parental cells at PD 20 and harvested on PD 160. Whole-mounted retinas (superior regions) were stained with PNA or antibodies against red/green opsin. PNA or red/green opsin positive cells are more in the retinas treated with CNTF devices (A and B than the fellow control retinas (C and D). Quantitative analysis of PNA or red/green positive cells in the superior retina showed significantly more cells in CNTF-treated retinas (446±80 and 310±46, respectively) than the controls (141±40 and 108±46, respectively) (E, mean ± SD, n = 3). Cone ERGs were recorded at PD 135 from eyes implanted at PD 20 with CNTF secreting or control devices. Representative b-waves from CNTF treated and control eyes are shown in panel F. The onset of flash is indicated by an arrowhead. The amplitudes of b-waves from CNTF treated retinas (32.46±10.02 µV) is significantly larger than from control retinas (17.70±4.78 µV) (G, mean ± SD, n = 3). Asterisk indicates *P*<0.05; double-asterisk indicates *P*<0.01 (Student *t*-test). Scale bar: 50 µm.

Long-term delivery of CNTF to the retina was achieved by using CNTF-secreting microdevices [35]–[37]. Animals were implanted with CNTF devices in the left eyes and control devices in the right eyes at PD 20. Eyes were collected at PD160, 140 days after implantation. Both PNA- and red/green-opsin-positive cells in the CNTF treated retinas (Fig. 8A and 6B, respectively) were significantly more than those in the control retinas of the fellow eyes (Fig. 8C and 8D, respectively). Quantitative analysis shows that density of PNA-positive or red/green opsin-positive cells in CNTF treated retinas is significantly more than in the control retinas (Fig. 8E).

In another experiment, preservation of cone function was assessed by ERG recording. At PD 30, rats were implanted with CNTF-secreting devices in the left eyes and devices containing the parental cells as controls in the right eyes. Cone ERGs were recorded at PD 135. The cone b-wave amplitude in CNTF-treated eyes is significantly larger than in control eyes (Fig. 8F and 8G). Thus, sustained delivery of CNTF helps to preserve the function of cones as well.

## Discussion

The present study demonstrates that the secondary cone degeneration starts with loss of COS as an early sign, whereas the actual cell death occurs much later. In the early stages of degeneration, cones without COS are not only alive and but are capable of regenerating their outer segments, indicating that at this time the degeneration process is reversible. Sustained delivery of CNTF preserves cone cells and their function.

The delay between loss of COS and cell death is likely in the time frame of about two months in this model. This is based on the finding that loss of COS is readily observable at PD 20; that retinas of S334ter-3 rats lack of CAR-positive cells in the PNA-negative areas at PD 90 (Fig. 3); and that the CNTF-induced regeneration of COS was dramatically decreased at PD 80 (Fig. 4). The two-month window is rather large considering the rapid rod degeneration (about 10 days) in these animals. Thus, secondary cone degeneration is a relatively slow process, which not only provides an opportunity to study the pathology of secondary cone degeneration, but also serves a model for pre-clinical assessment of potential treatments. Furthermore, this finding raises the possibility that a similar window may exist in patients with RP. Because of the slow progression of degeneration in RP patients, this delay in patients could be very long, which would provide a large window to allow therapeutic intervention to save cones.

The observation that CNTF stimulated regeneration of COS demonstrates that in the early stages of degeneration, cones are capable of regenerating COS under favorable circumstances and thus the degeneration is reversible. Obviously, this finding has strong clinical significance. COS are functional organelles that initiate the conversion of photons to neuronal signals. Without COS, cones lose their light-sensing function. Regeneration of COS therefore means that in the early stages of degeneration, cones may restore their light sensing capabilities and thus restore vision. In light of this finding, we may want to re-think current therapeutic strategies for RP. At present, the goal of RP treatment is to slow down or stop the degeneration. Instead, we perhaps want to think more about restoring and maintaining useful vision. It is possible that in RP patients, many degenerating cones retain the capability of regenerating COS. If that is the case, it may be possible to promote regeneration of COS and thus restore vision to some extent for those patients. Consistent with this notion is a recent finding in a Phase I clinical trial using CNTF for retinal degenerations, in which one patient with late-stage RP had a significant improvement of vision [38].

CNTF is a neurotrophic factor that has been shown consistently to protect rod photoreceptor from degeneration [4]–[12]. Our present study demonstrates that sustained delivery of CNTF helps cones to preserve COS and cone function for an extended period. These findings provide experimental evidence to support long-term sustained delivery of neurotrophic factors such as CNTF as a treatment strategy to benefit RP patients by preserving cone function and useful vision.

The exact cause of secondary cone degeneration is not clear, although a number of hypotheses have been proposed, as reviewed by Delyfer and co-worker [2]. These include the secondary loss of cones due to 1) toxic byproducts of dying rod photoreceptors; 2) the loss of structural support by the loss of rods; 3) remodeling as evidenced by new and abnormal synaptic connections after rod degeneration; 4) changes in the RPE and Müller cells due to rod degeneration; and 5) the loss of rod-derived cone viability factors (RdCVFs) that are produced by rods and are thought to support cone survival; loss of rods leads to loss of RdCVFs [39]. In addition to these possibilities, activation of microglia during rod cell loss could be a factor [40], [41]. Oxidative stress is also believed to cause secondary cone degeneration: the loss of rods leads to exposure to a relatively oxygen-rich environment that is detrimental to cones [42]–[45]. Yet another potential mechanism of cone cell degeneration following rod loss is prolonged starvation due to nutrient deprivation in cones [46]. While it is unclear whether one or more of these mechanisms results in secondary cone cell death, the problem is further complicated by the findings in the present study of the S334ter-3 rat suggesting that there are two geographically separated populations of cones in terms of their susceptibility to the degenerative pressure(s). One population includes cones in the PNA-negative areas, which are more susceptible to the degeneration pressure(s), and the other population that is less susceptible in the PNA-positive areas. The geographic distribution of cones must now be taken into consideration when studying cellular mechanisms of cone cell death.

Taken together, the present study demonstrated that an early sign of cones degeneration is the loss of COS. CNTF promotes regeneration of COS and protects cone from further degeneration. Long-term treatment with CNTF starting at an early stage of degeneration could be a viable strategy for preservation of vision for RP patients.

## Materials and Methods

### Ethics Statement

All procedures involving animals adhered to Association for Research in Vision and Ophthalmology Statement for the Use of Animals in Ophthalmic and Vision Research and were approved by the Institutional Animal Care and Use Committee of University of Miami, Miller School of Medicine.

### Animals and Intravitreal Injections

Homozygous transgenic rats (Sprague-Dawley background) carrying a murine rhodopsin mutant S334ter (line3) and wild-type Sprague-Dawley (Harlan Laboratories, Indianapolis, IN) rats were used in all experiments. The rate of photoreceptor degeneration in the homozygotes was similar to that observed in heterozygous animals [31]. Intravitreal injections were delivered through 33-gauge needles connected to 10-µl microsyringes (Hamilton, Reno, NV), as described previously [29]. One eye (left) of an animal was injected with recombinant human CNTF protein and the other with phosphate buffered saline (PBS). Eyes were collected 10 days after injection.

### Recombinant CNTF Protein

Expression and purification of recombinant CNTF protein were as previously described [29]. The open reading frame of human CNTF cDNA was cloned into an expression vector pQE30 (Qiagen, Valencia, CA) to generate plasmid pQE-CNTF. Recombinant human CNTF protein was expressed in E. coli (XL-blue, Stratagene, La Jolla, CA) and purified by immobilized-metal affinity chromatography on Ni-NTA Agarose columns (Qiagen, Valencia, CA) under native conditions. Eluted protein was buffer-exchanged to phosphate buffered saline (PBS). Purified protein was stored at −80°C.

### Implantation of CNTF Secreting Microdevices

CNTF secreting microdevices, provided by Neurotech USA (Lincoln, RI), were microtubes of 1.5 mm×0.5 mm in size, small enough for implanting into rat eyes. The wall of the microtubes was made of 90–100 µm thick semi-permeable hollow-fiber membrane. CNTF-secreting cells were encapsulated in the devices using the Encapsulated Cell Technology (ECT) [35]–[37]. The devices used in the experiments had a mean CNTF output of 0.8 ng/24 hr in culture. Control devices had parental cells with no CNTF-secreting capability.

For long-term studies, microdevices were implanted in the vitreous at postnatal day (PD) 20. To implant a microdevice, the sclera on the temporal side was exposed and an incision was made between the limbus and equator with a sharp #28 needle to reach the vitreous. A microdevice was put into the vitreous through the insertion at a 45° angle toward the posterior pole. The position of an implanted microdevice was confirmed visually through the pupil.

### Tissue Preparation and Immunohistochemistry

For whole-mounted retinas, animals were killed by CO_2_ inhalation and perfused with PBS. Eyecups were prepared by removing the cornea along the limbus and then the iris. Retina-lens preparations were obtained by first making a small cut at the edge of sclera toward the posterior pole. Then with a pair of forceps at each side of the cut, the sclera, along with the choroid and RPE (retinal pigment epithelium), was carefully torn open, and the sclera-choroid-RPE was peered off from the retina-lens to the base of optic nerve. The sclera-choroid-RPE was then cut off from the base of optic nerve. The resulted retina-lens preparations were post-fixed in cold 4% paraformaldehyde solution for 4 hours at 4°C.

For retinal cryo-sections, animals were killed by CO_2_ inhalation and perfused with PBS then 4% paraformaldehyde. Eyes were collected and the corneas and lenses were removed. The resulted eyecups were fixed in 4% paraformadehyde in 0.1 M phosphate buffer for 4 hrs and then equilibrated in 30% sucrose in 0.1 M phosphate buffer overnight at 4°C. Retinal sections (16 µm) were cut on a cryostat at −20°C, mounted on Super Frost Plus Gold slides (Fisher Scientific, Pittsburgh, PA), and air dried overnight at room temperature (RT).

Peanut agglutinin (PNA) was used to identify COS. For PNA staining, retina-lens preparations were incubated with FITC-conjugated (Vector, Burlingame, CA) or Alexa fluor 488-conjugate (Invitrogen) PNA. The lenses were removed and the retinas were flat-mounted on slides.

For immunostaining, retina-lens preparations or cryo-sections were incubated with anti-red/green opsin; anti-blue opsin (Santa Cruz Biotechnology); or anti-mouse cone arrestin (LUMIJ, a gift of Dr. Cheryl Craft of the University of Southern California), and visualized with Alexa Fluor 594 anti-goat IgG (Invitrogen, Carlsbad, CA) or Cy3 conjugated anti-rabbit antibody (Jackson immunoresearch lab, West grove, PA). For double-staining with PNA, Alexa fluor 488-conjugate PNA was added to the secondary antibody solution. All samples were examined by confocal microscopy.

### Quantification of PNA-Positive Cells

Confocal images from whole mount of retina were used to calculate the cone cell density (PNA or red/green opsin positive cells) in the superior retinas using software Volocity (Improvision, Waltham, MA). Data were expressed as mean±S.D.

### ERG Recording

Cone ERGs were recorded with a UTAS system (LKC Technologies, Gaithersburg, MD). Under Animals were anesthetized with intraperitoneal ketamine (80 mg/kg) and xylazine (4 mg/kg). Pupils were dilated with 0.1% atropine and 0.1% phenylephrine HCl. During the recording, animals were maintained at 35°C on a heated plate. A contact lens electrode with a platinum wire was placed on the cornea, a differential electrode under the skin of the forehead, and a ground wire electrode was placed under the skin close to the base of the tail. Both eyes were recorded simultaneously. Photopic ERG responses were elicited by 1 ms white flashes of 2.5 log cd·s/m^2^ generated by white LEDs in the Ganzfeld sphere of the UTAS system, with low white background illumination (30 cd·s/m^2^). Inter-stimulus intervals were 10 s. Each recording was an average of 10 responses.

### Statistic Analysis

Statistic analyses were performed using InStat (version 3.0, GraphPad Software Inc., San Diego, CA). Results were evaluated by ANOVA or Student's *t*-test for comparisons between experimental groups.
